# Assessment of motor function and nutritional status in children with spinal muscular atrophy treated with nusinersen after loading period in Western China: a retrospective study

**DOI:** 10.1186/s12883-023-03063-3

**Published:** 2023-01-23

**Authors:** Hua Yang, Qiuji Tao, Dan Li, Jie Yang, Qianyun Cai, Jing Gan, Shaoping Huang, Rong Luo

**Affiliations:** 1 Department of Pediatrics, West China Second University Hospital, Sichuan University, Chengdu, China; 2 Key Laboratory of Obstetric & Gynecologic and Pediatric Diseases and Birth Defects of Ministry of Education, Sichuan University, Chengdu, China; 3 Department of Pediatrics of neurology Nursing, West China Second University Hospital, Chengdu, China; 4 The Second Affiliated Hospital, Xi’an Jiaotong University, Xi’an, China

**Keywords:** Spinal muscular atrophy, Nusinersen, Motor function measure, Nutritional status, Children

## Abstract

**Background:**

Spinal muscular atrophy (SMA) is a progressive degenerative neuromuscular disease. Nusinersen, with its quick onset of action, can benefit patients early in the treatment course. However, there are currently no clinical studies regarding the improvement in motor function and nutritional status of patients after loading period treatment with nusinersen. Here, we aimed to determine the efficacy of nusinersen in improving motor function and nutritional status in children with SMA treated with nusinersen after loading period in Western China.

**Methods:**

In this retrospective study, data for all pediatric patients (aged < 18 years), with genetically confirmed diagnosis of SMA who were treated with nusinersen, were collected before initiation of treatment and after 2 months of treatment. We assessed motor function using standardized scales and nutritional status of patients with SMA as well as side effects of nusinersen.

**Results:**

Forty-six pediatric patients aged < 18 years were enrolled in this study. After 2 months of treatment, the motor function of patients with SMA type 1, 2, and 3 improved. The difference in Revised Upper Limb Module scores from M0 to M2 was significant in patients with SMA type 2 and 3 (*P* = 0.004, *P* = 0.042, respectively). The difference in Hammersmith Functional Motor Scale Expanded scores from M0 to M2 in patients with SMA type 2 was also significant (*P* = 0.000). No significant differences were found for Children’s Hospital of Philadelphia Infant Test of Neuromuscular Disorder (CHOP-INTEND), Hammersmith Infant Neurologic Examination-Part 2 (HINE-2), and 6-Minute Walking Test (6MWT) scores between M0 and M2, but the scores of CHOP-INTEND, HINE-2, and 6MWT were all increased after loading period treatment. The overall improvement in nutritional status was not statistically significant. No serious adverse effects were observed.

**Conclusions:**

Our study provides evidence for the efficacy and safety of nusinersen and the nutritional status of pediatric patients with SMA after the loading period treatment. Motor function of all patients improved after 2 months of loading period nusinersen treatment. Patients with a shorter disease duration showed better response to treatment. Careful surveillance of nutritional status is needed in patients with SMA.

## Background

Spinal muscular atrophy (SMA) is the most common neuromuscular disorder in children, and the leading cause of death among children under 2 years. The incidence rate in the surviving European and American populations is approximately 1/10,000, and the carrier frequency is 1/40–1/50 [1]. While there are no clear data on the incidence rate in China, the frequency of carriers in the Chinese population is approximately 1/42 [2].

SMA is an inherited neuromuscular disorder, mainly caused by homozygous mutations in the survival of motor neuron 1 (SMN1) gene on chromosome 5q. The resulting lack of SMN protein leads to degeneration of α⁃ motor neurons in the spinal cord and brain stem. The main clinical features of SMA include muscle weakness and muscular atrophy due to degeneration of α⁃ motor neurons. The *SMN2* gene, which differs in only five base pairs from SMN1, results in expression of a truncated SMN protein. *SMN2* is a key disease modifier of SMA phenotype and the copy number of *SMN2* inversely correlates with disease severity [3]. SMA is divided into five subtypes according to the age of onset and maximum motor function; with type 1, 2, and 3 being common during childhood.

Nusinersen was the first antisense oligonucleotide (ASO) to be approved by the United States Food and Drug Administration (FDA) for the treatment of SMA in children and adults on December 23, 2016. Nusinersen was subsequently approved in China in 2019. Nusinersen is designated as a rare disease drug that is administered intrathecally. It acts by promoting the inclusion of exon 7 in the SMN2 copies and increases the production of SMN protein [4].

As SMA is a progressive degenerative neuromuscular disease, the rapid onset of action of nusinersen provides benefit to patients early in the course of treatment. There have been few clinical studies on nusinersen and data on efficacy and safety were limited. Particularly, clinical studies on the improvement of motor function and nutritional status of patients after loading period treatment with nusinersen have not been reported. Therefore, we aimed to determine the efficacy of nusinersen in improving motor function and nutritional status and to document adverse events seen in pediatric patients with SMA. These data would provide valuable information to guide clinical decision making when considering nusinersen in this patient cohort.

## Methods

We collected data retrospectively for all pediatric patients with SMA who had received four doses of nusinersen within the ‘loading phase’ in the first 2 months of treatment at the Department of Pediatrics, West China Second University Hospital, Sichuan University, and The Second Affiliated Hospital of Xi’an Jiaotong University between October 2019 and March 2022.

The inclusion criteria were: (i) genetically confirmed diagnosis of SMA with a homozygous deletion of exon 7 or other mutations in SMN1 gene on chromosome 5q13, (ii) clinically confirmed diagnosis of SMA, (iii) age < 18 years, and (iv) patients had received four loading doses of nusinersen according to the dosing schedule of days 0, 14, 28, and 63.

The patients received intrathecal injections of nusinersen and was discharged after 24 hours of hospital observation. Adverse events (AEs) were documented based on medical interviews after lumbar puncture and nusinersen treatment. After discharge, reports of adverse reactions were followed up through telephone interviews and parental report.

### Clinical assessments

We retrospectively collected the following data: gender, family history of SMA, SMA subtypes, SMN2 copy number, age at symptom onset, age at genetic diagnosis, age at baseline functional assessment before treatment, disease duration, time from diagnosis to treatment, total lumbar punctures, evaluation of nutritional status, and adverse events. Motor development milestones were evaluated using different scales depending on the patient’s age and SMA subtypes at baseline and post treatment. The World Health Organization (WHO) motor milestones were used to assess gross motor development in children with SMA type 1 and SMA type 2 [5]. The modified Hammersmith Infant Neurologic Examination-Part 2 (HINE-2) was designed to evaluate different aspects of neurologic function in infants from 2 months to 2 years [6]. The Children’s Hospital of Philadelphia Infant Test of Neuromuscular Disorders (CHOP-INTEND) was used in all patients younger than 2 years and in all non-sitters [7]. For sitters, the Hammersmith Functional Motor Scales Expanded (HFMSE) protocol was used with the addition of the Revised Upper Limb Module (RULM) for those who could sit at a table [8, 9]. The 6-Minute Walking Test (6MWT) was used to evaluate the activity endurance of walkers [10]. Each of our patients received two to four motor function assessments mentioned above.

As scoliosis in patients with SMA could interfere with accurate measurement of body length, nutritional status of these patients was determined by Weight/age Z-scores (WAZ). WAZ were calculated using the WHO Anthro (Plus) software [11]. WAZ < –3 standard deviation (SD) was defined as severe underweight. WAZ < –2SD was considered as underweight. WAZ between –2SD and + 2SD was considered normal. WAZ > +2SD was considered overweight, and WAZ > +3SD was classified as severely overweight.

### Statistical analysis

Data with normal distribution were expressed as mean ± SD. Data with non-normal distribution were expressed as median (interquartile range [IQR]). Comparison between WAZ at M0 and M2 was performed using paired *t*-test. Comparison between disease duration and treatment response was performed using independent sample *t*-test. Comparison between motor results at M0 and M2 was performed using Wilcoxon Signed Rank Test. SPSS software version 23 was used for all statistical analyses, with significance set at *P* < 0.05.

## Results

### Patient demographics

**Fig. 1:**
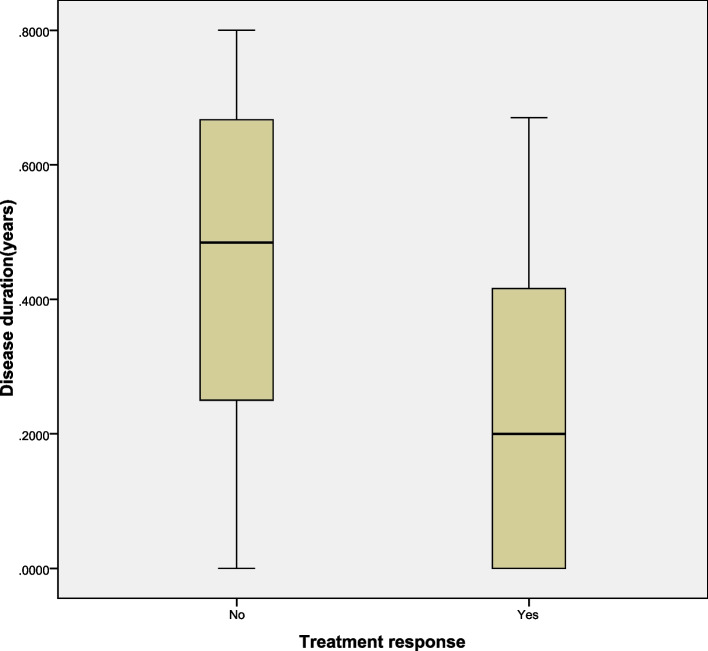
Disease duration as a predictor of gain in HFMSE after 2 months of treatment. A change of two or more points in HFMSE showed in the figure. In the group that experienced motor responses the median disease duration was 0.23 ± 0.05 years, compared to the median disease duration of 0.47 ± 0.08 years in the group of patients that remained stable or worsened (*P* = 0.013)

Between October 2019 and March 2022, 46 patients (20 boys and 26 girls) with genetically confirmed 5q-SMA were treated. Of these, eight were SMA type 1 (three boys, five girls), 31 were SMA type 2 (14 boys, 17 girls), and seven were SMA type 3 (three boys, four girls). SMN2 copy number was examined in 45/46 patients; the remaining patient was not examined for SMN2 copy number due to refusal of parents. None of the patient’s parents had consanguineous relatives. Four patients had positive family history of SMA, all of whom were female with SMA type 2. Two patients were twins and their elder sister was healthy. Another patient had an elder brother with SMA type 2, and her elder sister was healthy. The remaining patient had a younger sister with SMA type 2. Age at baseline functional assessment was different in SMA 1, 2, and 3. Mean age were 3.25 ± 2.19 years, 5.36 ± 4.09 years, and 5.87 ± 3.19 years, respectively. The age of genetic diagnosis was the youngest and the duration of disease was the shortest in patients with SMA type 1. The age of genetic diagnosis was the oldest and the disease duration was longest for SMA type 3. The age of genetic diagnosis and disease duration of SMA type 2 was between those for SMA type 1 and 3. In an exploratory analysis to identify potential predictors of gain in motor function after 2 months of treatment (defined as an increase of two or more points on the HFMSE), disease duration showed a significant difference as a predictor of increased HFMSE scores after 2 months of treatment, as shown in Fig. 1. Clinical characteristics are summarized in Table 1.

**Table 1:** Demographic and clinical variables in children with SMA

	SMA Type 1	SMA Type 2	SMA Type 3	Total
Number	8	31	7	46
Male/Female	3/5	14/17	3/4	20/26
SMN2 copy number				
Two copies	1	5	0	6
Three copies	7	26	6	39
Positive family history	0	4	0	4
Age at symptom onset (y)	0.5 (0.33, 0.68)	0.9 (0.7, 1.3)	2.5 (1.3, 3.0)	0.95 (0.7, 1.3)
Age at genetic diagnosis (y)	0.65 (0.5, 1.18)	1.3 (1.0, 1.6)	2.9 (1.8, 3.7)	1.3 (0.875,1.85)
Disease duration (y)	0.15 (0.03, 0.2)	0.3 (0.1, 0.6)	0.5 (0.1, 0.7)	0.25 (0.08, 0.53)
Time from diagnosis to treatment (y)	2.05 (0.35, 4.7)	2.3 (0.8, 6.0)	1.2 (1.0, 4.1)	1.95 (0.8, 4.88)
Baseline age of functional assessment (y)	3.25 (2.19)	5.36 (4.09)	5.87 (3.19)	5.07 (3.74)
Ventilation	2	2	0	4
Tongue fasciculations	8	31	6	45
Areflexia/hyporeflexia	8	31	7	46
Dysphagia	2	0	0	2
Scoliosis	3	12	2	17
Arthrogryposis	2	12	0	14
Spinal surgery	0	1	0	1
Lumbar punctures in total	32	125	28	185
Baseline WAZ	−1.22 (1.5)	−0.52 (1.39)	−0.06 (0.80)	−0.59 (1.36)
Post-treatment WAZ	−1.51 (1.16)	− 1.1 (1.81)	− 0.67 (1.2)	−0.91(1.44)
*P-*value*	0.32	0.008	0.051	0.01

*SMA* spinal muscular atrophy, *SMN2 *survival motor neuron 2, *WAZ *Weight /age Z-scores; Disease duration, a child’s age at genetic diagnosis minus the age at symptom onset; *P*-value*, *P*-value for baseline (M0), and post treatment (M2) WAZ

The age at initiation of treatment varied between 0.7 and 15.3 years. Only two of 46 patients (4.3%) had dysphagia but did not require a feeding tube. A total of four patients (8.7%) needed non-invasive ventilation (two with SMA type 1 and 2 with SMA type 2). All patients with SMA type 1 and 2 had tongue fasciculations, which was also seen in most patients with SMA type 3 (6/7).

Of the 46 patients, one with SMA type 3 had hyporeflexia, and the rest had areflexia. Scoliosis of varying severity was clinically and radiologically diagnosed in 17 patients (37.0%), most of whom had SMA type 2 (12/17). One female patient with SMA type 2 underwent scoliosis surgery due to severe scoliosis, with Cobb angle greater than 50 degrees. A total of 14 patients (30.4%) had arthrogryposis (two with SMA type 1, and 12 with SMA type 2) (Table 1).

### Motor capabilities

#### SMA type 1

Patients were examined using standardized motor scales before the initiation of treatment and after the fourth injection of nusinersen. The type of scale used depends on the age and motor function of the children. Of the eight patients with SMA type 1, six were evaluated using CHOP-INTEND and six were evaluated with HINE-2. The scores of CHOP-INTEND and HINE-2 increased by 1.0 and 0.5 points, respectively, compared to baseline motor function, but no significant differences were found for CHOP-INTEND and HINE-2 scores between M0 and M2 (*P* = 0.416, *P* = 0.416, respectively) (Table 2).

**Table 2:** Motor function of SMA patients before and after treatment

	SMA type 1		SMA type2		SMA type 3		
	CHOP-INTEND	HINE-2	RULM	HFMSE	RULM	HFMSE	6MWT (m)
Number	6	6	20	22	6	5	5
Baseline	19.5 (11.9)	4.0 (2.9)	15.8 (8.7)	12.5 (9.8)	29.3 (4.8)	44.2 (11.4)	199.9 (196.7)
Day 63	20.5 (12.1)	4.5 (3.6)	17.6 (9.3)	15.0 (10.6)	32.7 (2.7)	47.2 (11.6)	226.3 (236.5)
*P-*value	0.416	0.416	0.004	0.000	0.042	0.176	0.345

*CHOP-INTEND *Children's Hospital of Philadelphia Infant Test of Neuromuscular Disorder, *HINE-2 *modified Hammersmith Infant Neurologic Examination-Part 2, *RULM *Revised Upper Limb Module, *HFMSE *Hammersmith Functional Motor Scale Expanded, *6MWT* 6-Minute Walking test, *m* meters

#### SMA type 2

**Fig. 2:**
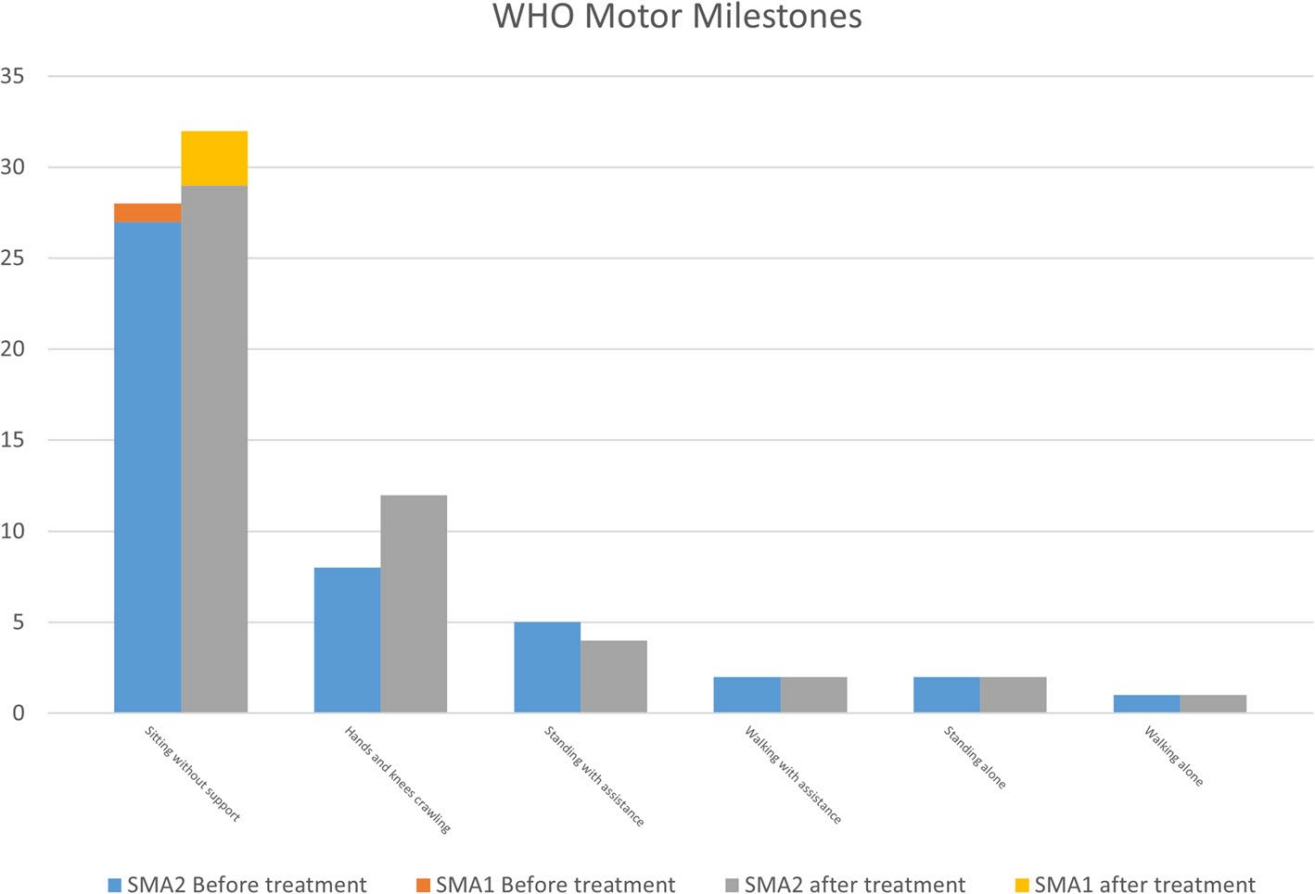
WHO Motor Milestones change in SMA type 1 (*n* = 8) and SMA type 2 (*n* = 30) patients before and after loading period treatment

Twenty-seven patients with SMA type 2 completed follow-up visits on day 63. 20 were evaluated using RULM and 22 were evaluated using HFMSE. The average motor performance improved after the fourth injection, as measured by RULM and HFMSE. RULM score improved by 1.8 points and HFMSE by 2.5 points, on average. From M0 to M2, the differences in RULM and HFMSE scores were significant (*P* = 0.004, *P* = 0, respectively). Eight patients with SMA type 1 and 30 with SMA type 2 were evaluated using the WHO motor milestones. The ability to sit alone and crawl was improved on day 63 (Fig. 2).

#### SMA type 3

**Fig. 3:**
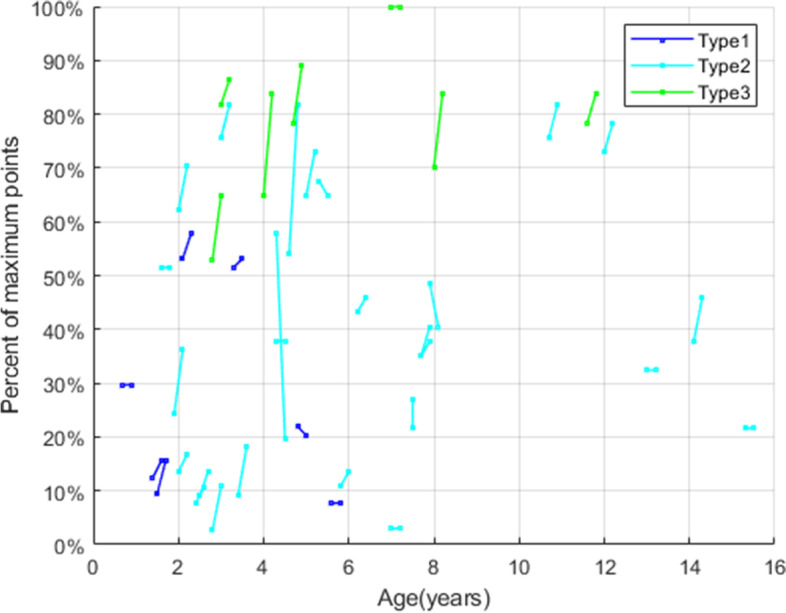
Longitudinal representations of percentages of maximum points on motor scales at M0 and M2 for all SMA subtypes

Seven patients with SMA type 3 completed follow-up visits on day 63. Six were evaluated using RULM, 5 using HFMSE, and 5 using the 6MWT. The average motor performance measured using RULM improved significantly by 3.4 points and by 3 points for HFMSE, after the fourth injection. The distance walked during the 6MWT improved in three patients, with an increase of 26.4 m. The difference in RULM score from M0 to M2 was significant (*P* = 0.042), but no significant differences were found for HFMSE and 6MWT score between M0 and M2 (*P* = 0.176, *P* = 0.345, respectively) (Table 2). The longitudinal representations of percentages of maximum points on motor scales including CHOP-INTEND, HINE-2, RULM, and HFMSE are presented on Fig. 3 for all SMA subtypes.

#### Nutritional status

**Fig. 4:**
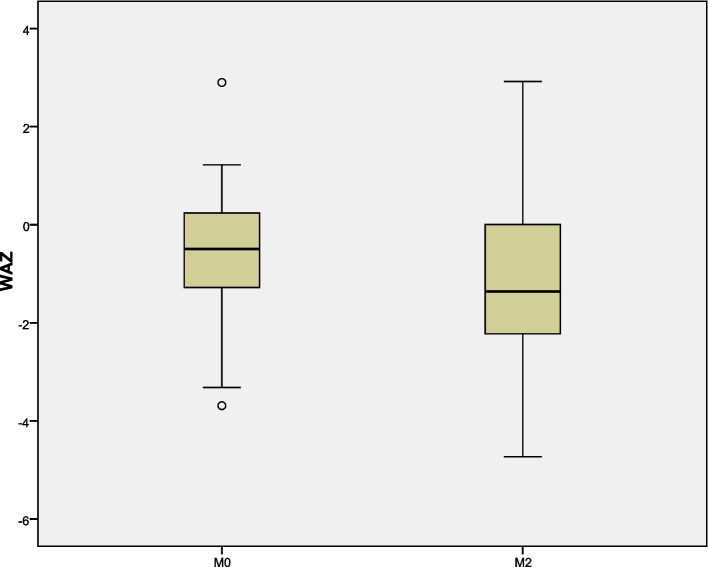
Comparison of values of WAZ at baseline (M0) and after the loading period (M2) treatment of nusinersen

**Fig. 5:**
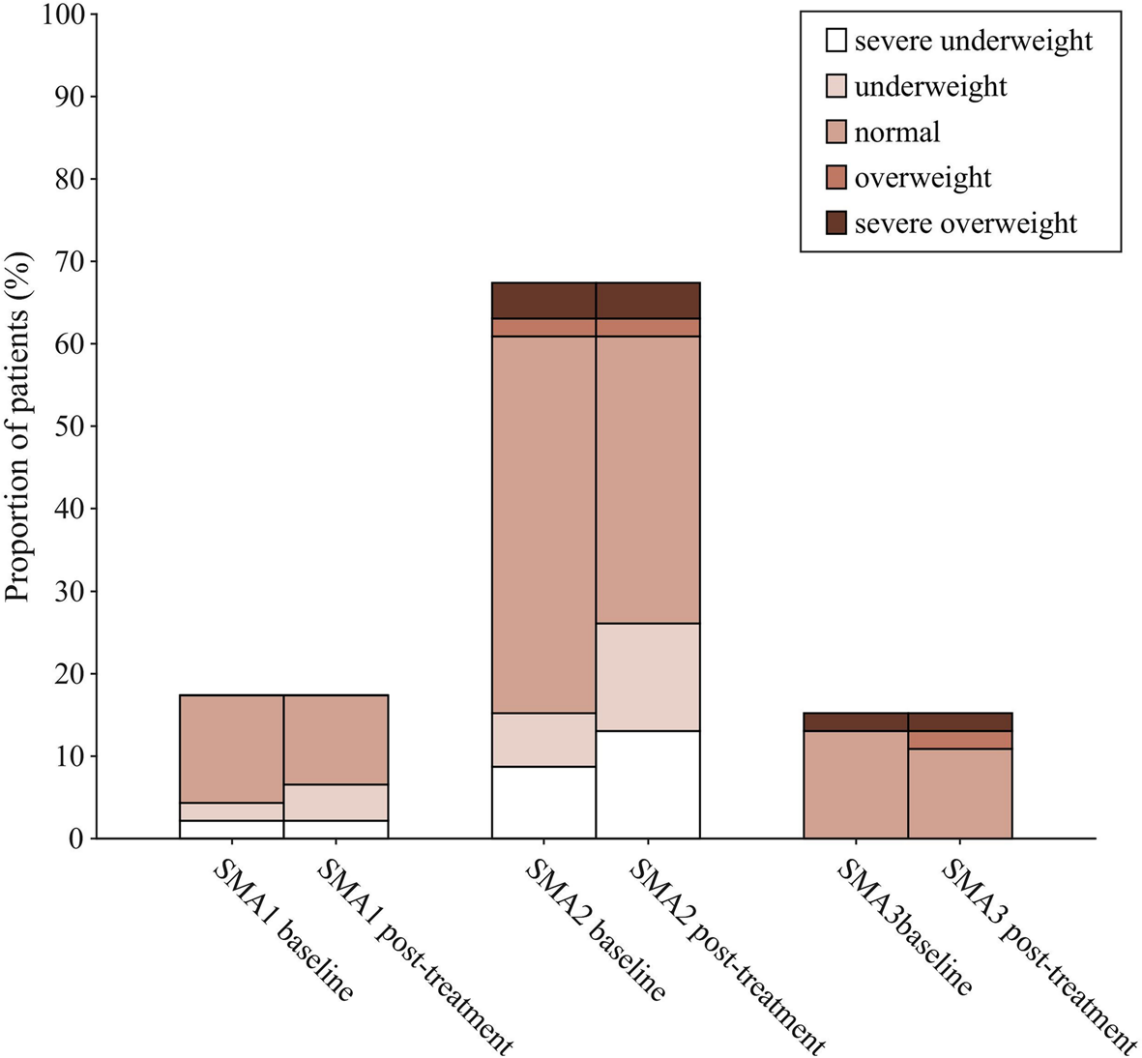
Change from M0 to M2 in nutritional status in SMA types 1, 2, and 3

Body weight of all patients with SMA were recorded before and after nusinersen treatment, and their nutritional status was determined by WAZ. Nutritional disorders were common in patients with SMA. The difference in WAZ from M0 to M2 was significant in patients with SMA type 2 (*P* = 0.008), but no significant differences were found in patients with SMA type 1 and SMA type 3 (*P* = 0.32, *P* = 0.051, respectively) (Table 1). The nutritional status by SMA types were presented on Figs. 4 and 5.

#### Adverse events

A total of 185 lumbar punctures were performed, and one failed in a patient with SMA type 2. The puncture failed because the puncture needle strayed into the blood vessel, which led to blood mixture in the cerebrospinal fluid. The patient delayed nusinersen treatment for 1 day, which was within the allowable delay time. Two patients with SMA type 2 received CT-guided intrathecal injections of nusinersen for severe scoliosis, and one of them had undergone spinal surgery. None of the patients had their treatment interrupted due to failure of lumbar puncture. There were 18 AEs related to the intrathecal administration of nusinersen. None were serious side effects, such as central nervous system infection, bleeding, paresis, hydrocephalus, and renal toxicity. Minor side effects were reported in 19/46 (41.3%) patients during the study period. Most of the side effects were observed after the first injection (11 patients). Eight patients reported side effects after more than one injection. The most common side effects were upper airway infection (seven patients) and vomiting (four patients) (Table 3).

**Table 3:** AEs after lumbar injections

	Day 1	Day 14	Day 28	Day 63
Total AEs	18	7	4	3
Total patients with AEs	11	5	3	3
Dizziness	1	1		
Weakness	1		1	
Back pain	2			
Fatigue			1	
Nausea	1	1		
Vomiting	3	1		
Diarrhea	1			
Abdomen pain	1			
Low fever	1	1		
Upper airway infection	4	2		1
Pneumonia	2			
Gastroenteritis				1
Myalgia Irritability	1	1		1
Subcutaneous hemorrhage			1	
Rash			1	

## Discussion

The availability of different therapeutic options has highlighted the need for reliable short-term and long-term data. A better understanding of the efficacy of each approach and the possible effects of combination therapies or therapeutic changes is needed. Here, we reported on the results of motor functional changes after 2 months of nusinersen treatment in patients with SMA.

A study on patients with SMA type 1 who were treated with nusinersen showed that HINE-2 scores were negatively correlated with disease duration [12]. In the ENDEAR study, 28% of patients gained ≥5 points in HINE-2 scores (M10) at a mean nusinersen initiation age of 5.4 months [13]. In another study on nusinersen treatment in patients with SMA type2 and type3, the improvements in HFMSE scores were smaller with longer disease duration [14]. In our study, SMA type 2 and type 3 who had a longer disease duration demonstrated a poor treatment response (a change of less than 2 points in HFMSE) Therefore, early diagnosis and shorter disease duration may be predictors of better treatment outcome with nusinersen.

In our study, no significant differences were found for CHOP-INTEND scores between M0 and M2. In contrast to the natural history of SMA type1 typically associated with a gradual decline in CHOP-INTEND scores [15], our data showed an increase of 1 point from M0 to M2. Szabó L et al. also found that the CHOP-INTEND scores improved by mean 3.4 points from baseline in patients treated with nusinersen. The change was statistically significant at the 5th injection and remained significant at the visit on day 307 [16]. A study in Italy reported that improvements in CHOP-INTEND increased gradually, and were more evident after 6 months, and a further but smaller increase was seen between 6 and 12 months [17]. We found that patients with SMA type 1 showed only a slight improvement in HINE-2, and no significant differences were found. In the ENDEAR study, significant improvements in HINE-2 scores were observed after 6 months [18]. A study of 50 patients with SMA type 1 found that the HINE-2 scores differed between baseline and 6 months but not between baseline and 2 months [19].

We assess the motor function of patients with SMA type 2 using RULM and HFMSE scale. Previous data on natural history of the disease had shown that the point of slope change in RULM was 5.8 years in patients with SMA type 2 [20]. In our study, the baseline age for motor function assessment in patients with SMA type 2 was 5.36 years, and RULM scores increased by 1.8 points on average after 2 months of nusinersen treatment. The difference in RULM score was significant from M0 to M2. Mercuri E et al. observed an increase by a mean of 1.31 points in RULM 2 months after treatment in patients who were unable to walk [21]. Similarly, Jochmann et al. found that RULM scores increased in five of the seven patients treated with nusinersen after 2 months [22]. Previous data on natural disease history had shown that the peak of abilities gained on the HFMSE scale occurred before the age of 5 years. The highest number of lost abilities was found in the group aged between 5 and 13 years [23]. In our study, there was an increase of 2.5 points in HFMSE scores after 2 months of nusinersen treatment, and the difference was significant from M0 to M2 in patients with SMA type 2. In the CHERISH study, Mercuri et al. found that the HFMSE scores improved after 3 months of nusinersen treatment [24]. On the other hand, Jochmann et al. found that HFMSE scores increased in 2 patients, remained unchanged in five patients, and decreased in one patient after 2 months of treatment among seven treated with nusinersen [22]. Our findings are consistent with those of Elsheikh B et al., in which HFMSE score showed a mean difference from baseline of 2.77 points at 2 months in 23 ambulant patients, and another study showing that the mean scores increased by 0.77 point in non-ambulatory patients [21, 25]. Szabó et al. found that HFMSE scores in SMA type 2 patients increased from baseline to 2 months after nusinersen treatment [16]. In our study, we observed slight improvement in the WHO milestone in terms of sitting alone and crawling with hands and knees on day 63. There are few studies on the WHO motor milestones in SMA children after nusinersen treatment, and further studies are needed in the future.

Montes J et al. found an overall decline on the 6MWT over time in a natural history study of ambulatory function in patients with SMA type 3 [26]. In our study, no significant differences were found for 6MWT score between M0 and M2, but we found a meaningful increase of 26.4 m in the 6WMT of patients with SMA type 3. This is consistent with a study by Elsheikh et al. that found a 19.49-m increase in 6WMT in ambulatory patients with SMA type 3 after 2 months of treatment with nusinersen [25]. A study in patients with SMA in Hungary also found improvement in the 6WMT after 63 days of treatment [16]. In addition, there were significant increase in RULM scores in patients with SMA type 3. Taken together, our results showed that motor function of SMA patients improved overall after loading period of nusinersen treatment.

To date, only a few studies with small samples have investigated the nutritional aspects of patients with SMA and few used a standardized protocol to assess growth patterns. In our study, none of the patients underwent gastrostomy despite their low body weight, and only two patients had a history of swallowing problems. Our study also found that nutritional disorders were prevalent in children with SMA. In a natural history study of 102 patients with SMA type 2, 28% of them had a body mass index/age z-scores <− 2 SD, indicating that close monitoring of weight change is needed in patients SMA type 2 [27]. A study on motor function in children with SMA type 1c and type 2 who were treated by nusinersen showed that body weight was improved in all patients at M14 (six injections) compared with M0 [28]. In our study, a few patients showed improved nutritional status after proper nutritional management. However, due to the short follow-up time, the overall improvement in nutritional status was not statistically significant. Further studies with longer follow-up duration, which include assessments of chewing and of lean/fat body mass, will help to better understand the possible mechanisms underlying weight issues in these patients.

With regard to the safety profile, 41.3% of patients reported side effects during the study period, but none were severe. Most AEs occurred after the first injection, and the most common side effects were symptoms of infections, such as upper airway infection, and post-puncture symptoms, such as vomiting, pneumonia, and gastroenteritis. No hydrocephalus or other significant drug-specific side effects were observed. No treatment was terminated due to side effects.

## Conclusions

In conclusion, we showed that nusinersen was effective against SMA type 1, 2, and 3 after 2 months of loading period treatment. Motor function in patients with SMA improved after the loading phase. In addition, patients with shorter disease duration showed better response to treatment. Our results confirmed that careful surveillance of nutritional status is needed in patients with SMA. In the present study, no significant drug-related side effects were observed after the administration of the fourth injection. Our findings on the outcomes of patients with SMA treated with nusinersen can provide a better understanding of the disease, contribute to improved clinical and nutritional management of patients, and add to the assessment of disease-modifying treatment effects on SMA.

## Data Availability

Data are available upon reasonable request to corresponding author.

## Abbreviations

6MWT: 6-Minute Walking Test
AE: adverse event
ASO: antisense oligonucleotide
CHOP-INTEND: Children’s Hospital of Philadelphia Infant Test of Neuromuscular Disorder
FDA: Food and Drug Administration
HFMSE: Hammersmith Functional Motor Scales Expanded
HINE-2: Hammersmith Infant Neurologic Examination-Part 2
IQR: interquartile range
m: meters
RULM: Revised Upper Limb Module
SD: standard deviation
SMA: Spinal muscular atrophy
SMN1: survival of motor neuron 1
WAZ: Weight/age Z-scores
WHO: World Health Organization
